# The number of methylated CpG sites within the *MGMT* promoter region linearly correlates with outcome in glioblastoma receiving alkylating agents

**DOI:** 10.1186/s40478-021-01134-5

**Published:** 2021-03-04

**Authors:** Sebastian Siller, Michael Lauseker, Philipp Karschnia, Maximilian Niyazi, Sabina Eigenbrod, Armin Giese, Joerg-Christian Tonn

**Affiliations:** 1 Department of Neurosurgery, University Hospital, LMU Munich, Marchioninistrasse 15, 81377 Munich, Germany; 2 German Cancer Consortium (DKTK), Partner Site Munich, Pettenkoferstrasse 8a, 80336 Munich, Germany; 3 Institute for Medical Information Processing, Biometry and Epidemiology, LMU Munich, Marchioninistrasse 15, 81377 Munich, Germany; 4 Department of Radiation Oncology, University Hospital, LMU Munich, Marchioninistrasse 15, 81377 Munich, Germany; 5 Center for Neuropathology and Prion Research, University Hospital, LMU Munich, Feodor-Lynen-Strasse 23, 81377 Munich, Germany

**Keywords:** Glioblastoma, *MGMT* promoter, Methylation status, Temozolomide, Linear correlation, Methylation-specific polymerase-chain-reaction, MSP, Sanger sequencing

## Abstract

*MGMT*-promoter methylation is associated with favorable outcome in glioblastoma. The aim of this study was to determine whether the absolute number of methylated Cytosine-Guanine-dinucleotide-(CpG-)sites within the DMR-2 island of the *MGMT*-promoter may correlate with outcome in a qualitative or quantitative fashion. In a cohort of newly diagnosed glioblastoma patients treated with stereotactic biopsy or open tumor resection plus concomitant chemoradiotherapy, we assessed *MGMT*-promoter methylation by methylation-specific polymerase-chain-reaction (MSP). Methylation of the CpG-sites 74–98 within the *MGMT*-promoter region was additionally analysed by Sanger sequencing, and the total number of methylated CpG-sites was correlated with outcome using proportional hazards models. 215 patients with glioblastoma were identified and stratified per MSP (positive: 53%, negative: 47%). Among MSP-positive tumors, hierarchical clustering identified three subgroups with different methylation rates (median: 80% vs. 52% vs. 47%), indicating a site-dependent methylation propagation. The methylation status of a given CpG-site indicated a neighborhood-dependent methylation propagation. Survival was linearly associated with the cumulative number of methylated CpG-sites. This was particularly true in patients who received at least one adjuvant cycle of temozolomide. Notably, all CpG-sites analyzed contributed similarly to effect size; this enabled a further predictive substratification of MSP-positive tumors with median OS ranging from as low as 17.1 months (< 18 methylated CpG-sites) to as high as 26.2 months (≥ 18 methylated CpG-sites) in the overall cohort. All in all, total number of methylated CpG-sites may correlate with outcome in a linear fashion. Such analysis may therefore add further predictive value to conventional methods of determining the *MGMT*-promoter status.

**Supplementary Information:**

The online version contains supplementary material available at 10.1186/s40478-021-01134-5.

## Introduction

Glioblastoma is the most frequent primary brain tumor with a devastating natural history [23]. Surgical treatment followed by combined chemoradiotherapy and maintenance chemotherapy represents the ‘standard of care' in such tumors [32]. Epigenetic silencing of *O*-6-methylguanine-DNA methyltransferase (*MGMT*) by promoter methylation has been shown to be strongly associated with response to chemotherapy with alkylating agents [12, 27]. Hence, numerous clinical trials have stratified patients according to MGMT promoter methylation status [12, 14, 27].

Although MGMT promoter status has been shown to be of relevance with respect to treatment and outcome in glioblastoma patients, a consensus on methods and cutoff values to determine *MGMT* promoter methylation remains to be defined. Methylation-specific polymerase chain reaction analysis (MSP) [7, 12, 27] and pyrosequencing are among the most frequently used methods [5, 11, 21, 22]. Such methods usually analyze only a small number of the Cytosine-Guanine dinucleotide (CpG) sites within the differentially methylated region-2 (DMR-2) island of the MGMT promoter region, and results are typically reported either as ‘methylated’ or ‘unmethylated’ by laboratory core facilities [2, 19]. However, such approaches may miss the potential role of the patient’s individual CpG methylation pattern, the effect of quantitative differences in methylation, and cases of “grey zone methylation” as recently proposed [13, 24]. It is therefore unclear whether there might be a correlation between outcome and number of methylated CpG sites, particularly also those sites which are not analyzed by standard techniques like MSP.

In the present study, we describe a large cohort of patients with histologically verified glioblastoma treated at a single academic neuro-oncology center with standard of care including alkylating chemotherapy, where both MSP and Sanger sequencing (Sseq) were applied in parallel for MGMT promoter methylation testing and evaluated independently. Since, Sseq enables analysis of the individual methylation status of 25 single CpG sites (including those 9 CpG sites covered by MSP) located in the DMR-2 island and downstream of that [2], we aimed to elucidate the above mentioned role of the individual CpG site methylation profile on outcome and help to improve prognostic/predictive stratification of glioblastoma patients for personalized treatment concepts as compared to current standard approaches.

## Material and methods

### Patients

The institutional database of the Center for Neuro-Oncology at the University Hospital of the LMU Munich was searched for all adult patients with a de-novo histopathological diagnosis of a supratentorial glioblastoma consecutively treated with radiotherapy (RT) plus temozolomide (TMZ) between April 2005 and June 2015. The study was reviewed and approved by the local Institutional Review Board (IRB) of the Ludwig-Maximilian-University Munich (approval number 703/16), and a waiver of consent was issued by the IRB.

### Treatment

Patients underwent either open tumor resection (OTR) or stereotactic biopsy according to the recommendations of our interdisciplinary tumor board. The extent of OTR was determined by postoperative magnetic resonance imaging (MRI) provided within 72 h after surgery, and scored according to the study of Stummer et al. [26] either as gross-total tumor resection (GTR, no residual contrast enhancement in post-contrast T1-weighted sequences) or subtotal tumor resection (STR, any contrast enhancement with a volume of more than one voxel in the post-contrast T1-weighted images). Patients were scheduled to receive combined chemoradiotherapy (RT/TMZ) within 3 weeks upon histopathological diagnosis per ‘standard of care’ in accordance to the EORTC 22981/26981 protocol [28], and adjuvant TMZ up to 6 cycles was initiated within 6 weeks after chemoradiotherapy as consolidation per European guidelines whenever possible [30]. None of our patients > 70 years was treated with hypofractionated radiotherapy as this therapeutic approach was incorporated in our institutional practice after the recruitment period for this study [20]. Patients who refused or had contraindications for combined chemoradiotherapy received TMZ alone.

### Follow-up imaging

Gadolinium contrast-enhanced MRI was performed six weeks after completion of RT/TMZ and in 3 months-intervals thereafter or in any case of clinical deterioration. Treatment response as well as disease progression was assessed by our interdisciplinary tumor board according to the RANO criteria [31]. In case of diagnostic uncertainties, *O*-(2-[18F]fluoroethyl)-1-tyrosine (18F-FET) positron emission tomography (PET) with or without subsequent stereotactic biopsy was provided.

### Integrative diagnosis

Tissue specimens of all patients were reviewed according to the 2016 WHO classification of central nervous system tumors [17]. Isocitrate dehydrogenase 1 and 2 (IDH1 and IDH2) mutations were examined using pyrosequencing as previously described [6].

### Isolation of nucleic acids

DNA was isolated from each tumor specimen using the QIAamp® DNA Micro Kit (Qiagen, Hilden, Germany). DNA isolation from normal blood lymphocytes was performed either using magnet-based techniques (QuickPickTM gDNA, Bionobile, Turku, Finland) or the QIAmp DNA Blood Kit (Qiagen, Hilden, Germany). The quantity and purity of DNA were assessed using the NanoDrop® ND-1000 Spectrophotometer (NanoDrop, Wilmington, NC). DNA recovery from each stereotactic biopsy sample amounted to around 30–60 μg/l.

### Bisulfite modification of DNA

The bisulfite conversion reaction was performed with a total of 200–400 ng DNA by use of the EpiTect® Bisulfite Kit (Qiagen, Hilden, Germany). In this reaction, all cytosines except for their methylated counterparts are converted to uracil. For the detection of promoter hypermethylation of the *MGMT* gene, both MSP and Sanger sequence analysis were performed using bisulfite-modified DNA.

### MGMT promoter methylation: methylation-specific PCR

**Fig. 1:**
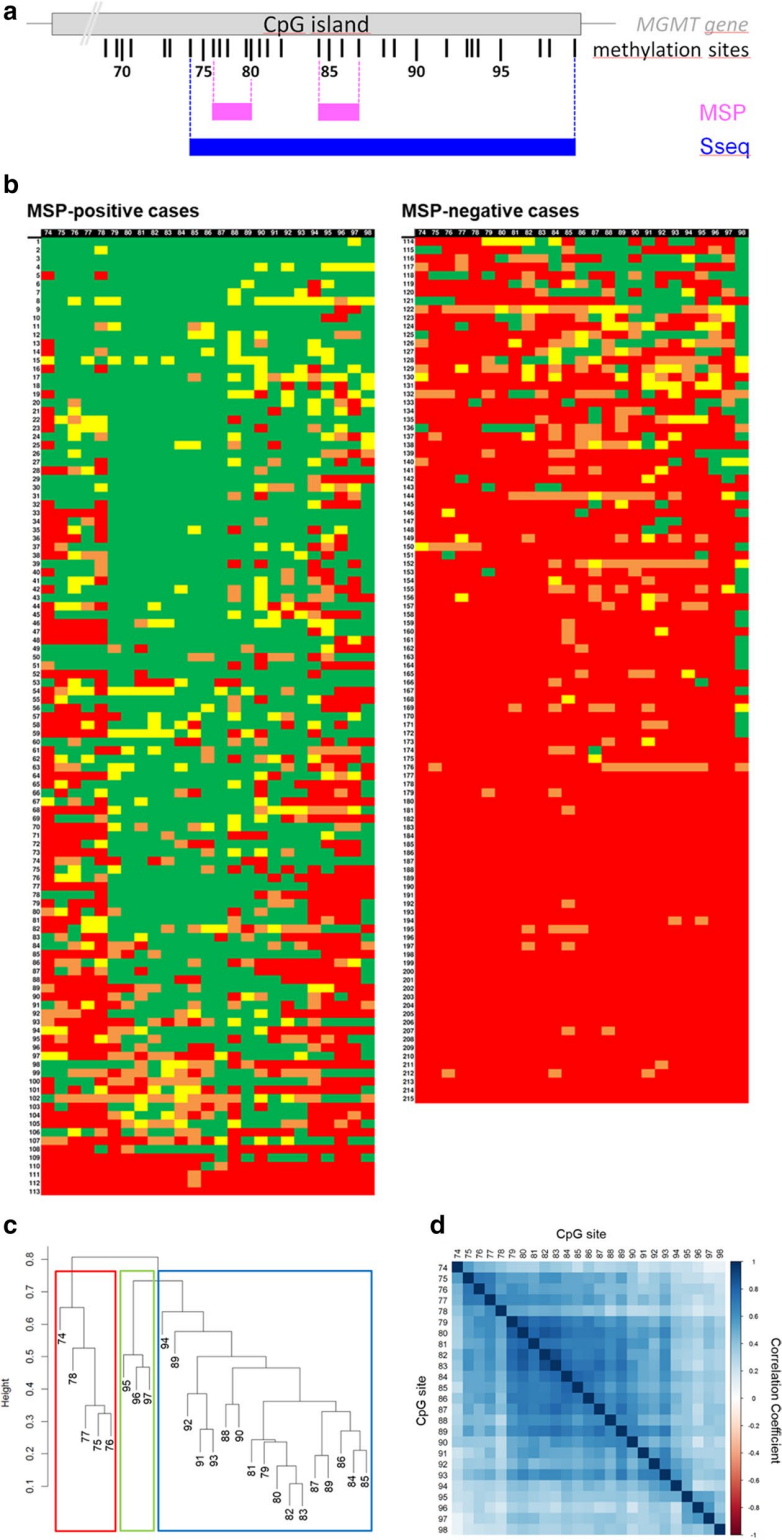
(**a**) Graphical representation of the MGMT 5′-CpG island methylation patterns detected by Sanger sequencing in 113 MSP-positive (left side) and 102 MSP-negative (right side) glioblastoma patients. The ordinate represents the case numbers, while the top abscissa represents the numbering of the MGMT promoter CpG residues investigated. The 25 CpG sites map between nucleotides 46,922 and 47,124 according to GenBank accession number AL355531. CpG sites were classified as ‘strongly methylated’ (ratio of C/T-peak > 1.00; green rectangles) and ‘partially methylated’ (ratio of C/T-peak 0.50–1.00; yellow rectangles), while unmethylated CpG sites were subdivided in ‘weakly methylated’ (ratio of C/T-peak 0.10–0.49; orange rectangles) and ‘non-methylated’ (ratio of C/T-peak < 0.10; red rectangles). (**b**) Hierarchical clustering of the 25 analyzed CpG sites of the MGMT promoter gene (CpG sites 74–98) using the Jaccard distance and the maximum linkage criterion. Three distinct clusters (red, green and blue colour) can be found. (**c**) Graphical representation of methylation correlations between the different CpG sites (abscissa and ordinate). Dark blue squares display high positive and dark red squares high negative intercorrelations, while white squares display no intercorrelations

For MSP, two pairs of primers encompassing CpG sites 76–80 and 84–87 (Fig. 1a) specific for either the methylated or the unmethylated *MGMT* promoter region were used as previously described [8]. Tumors were graded as ‘methylated’ or unmethylated’ as described by Grasbon-Frodl et al. [10].

### MGMT promoter methylation: Sanger sequence analysis of bisulfite-modified DNA

Sequencing and data analysis was performed as previously described [6, 10]. In brief, a 316 base pairs (bp) PCR product encompassing 25 CpG sites of the *MGMT* promoter (CpG sites 74–98; Fig. 1a) was obtained using the primers described by Moellemann et al. [22].

The respective CpG sites were classified as ‘methylated’ if they were ‘strongly methylated’ (ratio of cytosine/thymine [C/T]-peak > 1.00) or ‘partially methylated’ (ratio of C/T-peak 0.50–1.00), while unmethylated CpG sites comprised ‘weakly methylated’ (ratio of C/T-peak 0.10–0.49) and ‘non-methylated’ (ratio of C/T-peak < 0.10) CpG sites. Using these cutoff values has been shown to provide an easy classification with very high inter-rater reliability [6]. The total number of methylated CpG sites was calculated for each patient.

Raters were blinded for the clinical outcome data. MSP and Sseq were applied in parallel and evaluated independently.

### Statistical analysis

All statistical analyses were performed for complete datasets with R 3.4 (Comprehensive R Archive Network, CRAN) by an experienced biostatistician (ML) in cooperation with the neuro-oncological team (SS, JCT)**.** The reference point of this study was the date of surgery. Endpoints were progression-free survival (PFS), post-recurrence survival (PRS) and overall survival (OS). Patients were followed until death from any cause, or censored at day of last follow-up. Database closure was December 2018. Survival data were analyzed with the Kaplan–Meier method. For comparative analyses the log-rank test was used. Prognostic factors were obtained from proportional hazards models (Cox regression models). Multiple proportional hazards models for all CpG sites were fit by component wise likelihood based boosting [3]. Martingale residuals were plotted to assess the coherence between the total number of methylated CpG sites and survival hazards [29]. Hierarchical clustering of CpG sites was done using the Jaccard distance and the maximum linkage criterion [16]. The Bonferroni–Holm correction was used in case of multiple testing [15]. Correlation analysis was performed using the Spearman and Pearson correlation coefficient testing. *p* values below 0.05 were considered statistically significant.

## Results

A total of 215 consecutively treated patients with IDH1/2 wild-type tumors were encountered. Patients´ baseline characteristics are summarized in Table 1. OTR and biopsy were done in 100 (46.5%) and 115 (53.5%) patients, respectively; there was no significant difference in the proportion of patients with OTR versus biopsy between the subgroups of patients harboring MSP-positive or -negative tumors. RT/TMZ was provided in all patients afterwards. Biopsied patients were significantly older (63 vs. 58 years, *p* < 0.01), had worse clinical pretreatment status (median Karnofsky Performance Status [KPS]: 80 vs. 90, *p* < 0.01), and more often deep seated or multifocal tumor location (49.6% vs. 19.0%, *p* < 0.01).

**Table 1:** Clinical characteristics

Gender	
Male/female, (n/n, %/%)	142 (66.0)/73 (34.0)
Age (years)	
Median (range)	60 (17–86)
KPS	
Median (range)	80 (40–100)
Tumor side	
Left/right (n/n, %/%)	117 (54.4)/98 (46.6)
Tumor location	
Lobar/deep^a^/multifocal (n/n/n, %/%/%)	139 (64.7)/20 (9.3)/56 (26.0)
Surgical treatment	
GTR (n, %)	63 (29.3%)
STR (n, %)	37 (17.2%)
Biopsy (n, %)	115 (53.5%)

^a^Deep seated = not lobarly located (e.g. thalamus or basal ganglia)

According to MSP, 113 tumors (52.6%) were graded as MSP-positive and ‘methylated’. *MGMT* promoter methylation maps of the study population as determined by Sseq are given in Fig. 1b. Methylation patterns were heterogeneous between the individual patients, and CpG sites at the boundaries of the analyzed promoter island were found to be less often methylated. Patients with MSP-positive (− negative) tumors exhibited a wide range of cumulative numbers for methylated CpG sites [8–24 (median: 18) vs. 0–13 (median: 1)]. Generally, patients with MSP-positive (− negative) tumors showed higher (lower) cumulative numbers of methylated CpG sites (median: 18 vs. median: 1). Moreover, the cumulative number of methylated CpG sites within the MSP-primer region (CpG sites 76–80 and 84–87) were positively inter-correlated with the cumulative number of methylated CpG-sites outside the MSP-primer region (CpG sites 74, 75, 81–83 and 88–98) (Pearson correlation coefficient: 0.876; *p* < 0.001).

Among MSP-positive tumors, hierarchical clustering identified three subgroups with different methylation rates (Fig. 1c). Methylation level was highest in CpG sites 79–> 94 plus CpG site 98 (cluster I; median methylation rate: 80%), intermediate in CpG sites 74– > 78 (cluster II; median methylation rate: 52%), and lowest in CpG sites 95– > 97 (cluster III; median methylation rate: 47%). The differences in methylation levels between each of the clusters were statistically significant (*p* < 0.01). Methylated CpG sites were inter-correlated among themselves: CpG sites were more likely to be methylated when the bordering CpG site was also methylated (i.e. neighborhood-dependent methylation propagation) (Fig. 1d).

### Outcome and markers of outcome in the overall cohort

**Fig. 2:**
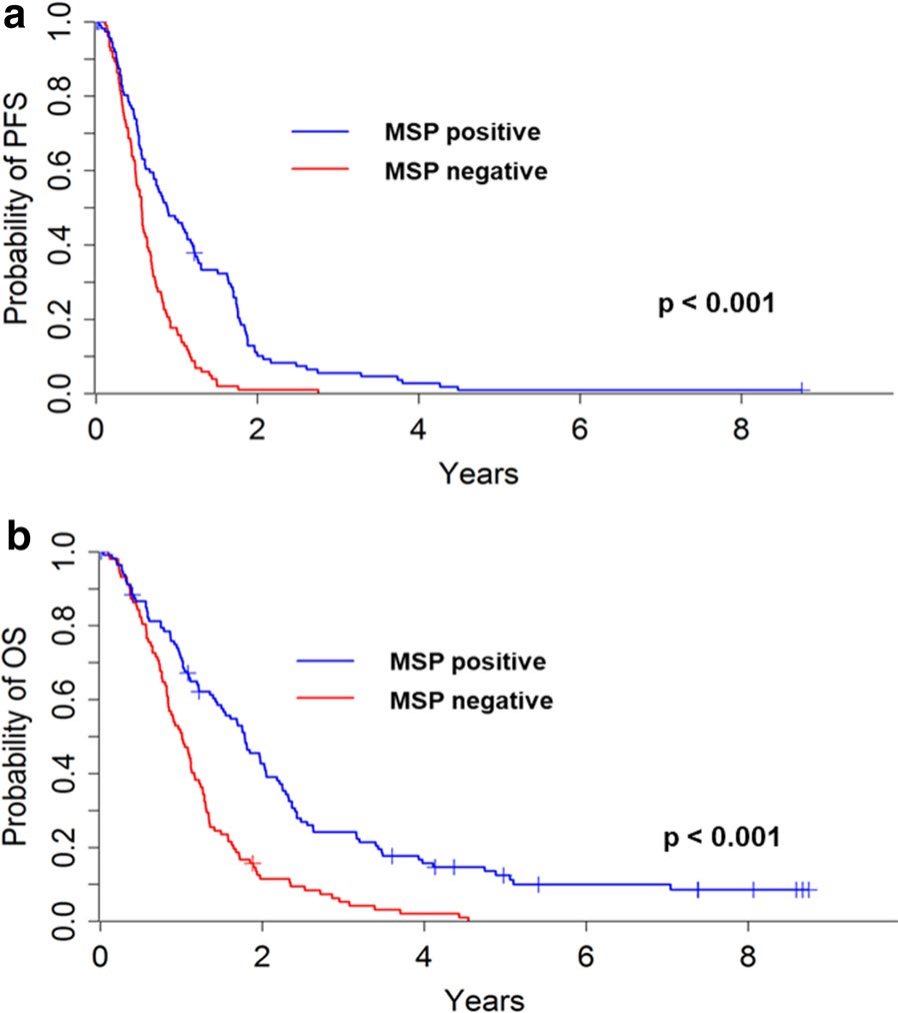
Kaplan–Meier curves of (**a**) progression-free and (**b**) overall survival for 215 glioblastoma patients stratified for MSP-positive (blue colour) or MSP-negative (red colour) tumors

At the time of last follow up, 211 patients experienced progressive disease and 199 patients were deceased. Death was tumor-related in all patients. Median PFS and OS were 7.9 and 14.9 months, respectively. Median survival for MSP-positive (MSP-negative) tumors was 21.4 (12.1) months (*p* < 0.001; Fig. 2a). Progression free survival was also highly divergent (Fig. 2b).

**Fig. 3:**
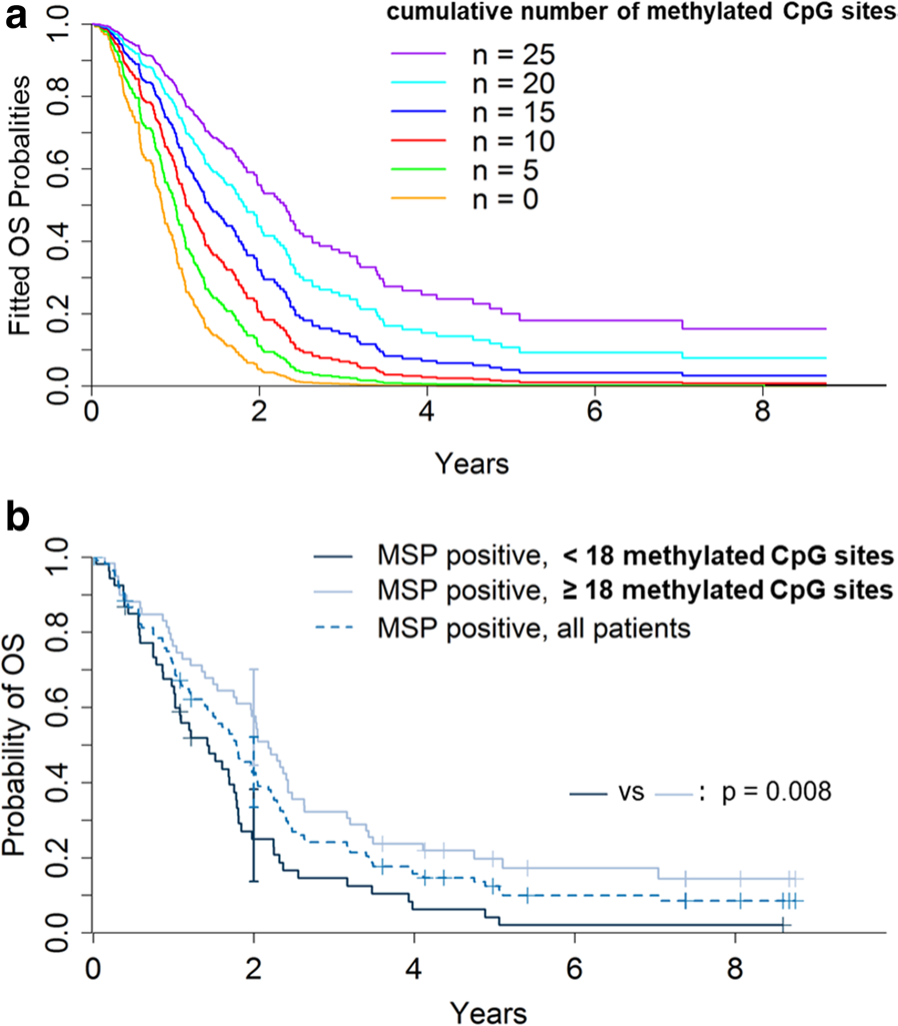
(**a**) Fitted probabilities according to the stratified Cox proportional hazards model of overall survival (OS) for 215 glioblastoma patients with regard to the cumulative number [n] of ‘methylated’ CpG sites (violet: n = 25, light blue: n = 20, blue: n = 15, red: n = 10, green: n = 5, orange: n = 0). (**b**) Kaplan–Meier curves of overall survival for 113 glioblastoma patients with MSP-positive tumors (dotted blue line) substratified for the cumulative number [n] of ‘methylated’ CpG sites (light blue line: n ≥ 18, dark blue line: n < 18)

Uni- and multivariable prognostic modelling are summarized in Table 2. On univariate analysis, the cumulative number of methylated CpG sites, MSP-positivity, younger age, and OTR were associated with increased PFS and OS. Additionally, a pre-treatment KPS ≥ 80 and a lobar tumor location were prognostic for favourable outcome. On multivariable models, the cumulative number of methylated CpG sites, younger age, and OTR retained their relevance as positive outcome markers on PFS/OS. Of note, the significance of the MSP status was lost in multivariable analysis. The adjusted hazard ratios for each methylated CpG site for disease progression and death were 0.94 (95% CI 0.93–0.96) and 0.94 (95% CI 0.92–0.96), respectively. The respective adjusted fitted survival curves are displayed in Fig. 3a with median OS ranging from as low as 10 months (0 methylated CpG sites) to as high as 28 months (25 methylated CpG sites).

**Table 2:** Prognostic factors

*Univariable*		
CpG sites		
Per methylated site in Sseq	0.95 (< 0.01/0.93–0.97)	0.95 (< 0.01/0.94–0.97)
Age (years)		
Per year	1.02 (< 0.01/1.00–1.03)	1.02 (< 0.01/1.01–1.04)
KPS		
<80 versus ≥ 80	2.00 (< 0.01/1.36–2.94)	2.99 (< 0.01/2.01–4.45)
Tumor side		
Left versus right	1.25 (n.s./0.95–1.64)	1.16 (n.s./0.87–1.53)
Tumor location		
Lobar versus other	0.90 (n.s./0.68–1.20)	0.82 (n.s./0.61–1.1)
MSP status		
Positive versus negative	0.44 (< 0.01/0.33–0.60)	0.45 (< 0.01/ 0.34–0.61)
Surgical treatment		
OTR versus biopsy	0.64 (< 0.01/0.48–0.86)	0.59 (< 0.01/ 0.45–0.80)
*Multivariable*		
CpG sites		
Per methylated site in Sseq	0.96 (< 0.01/0.93–0.99)	0.95 (< 0.01/0.92–0.99)
Age (years)		
P er year	1.02 (< 0.01/1.01–1.04)	1.03 (< 0.01/1.02–1.05)
KPS		
< 80 versus ≥ 80	1. 94 (n.s./1.30–2.91)	2.88 (< 0.01/1.90–4.36)
Tumor location		
Lobar versus other	1.04 (n.s./0.74–1.45)	1.08 (n.s./0.76–1.53)
MSP status		
Positive versus negative	0.77 (n.s./0.44–1.34)	0.68 (n.s./0.37–1.26)
Surgical treatment		
OTR versus biopsy	0.75 (n.s./0.54–1.03)	0.63 (< 0.01/0.44–0.89)

Survival for those MSP-positive tumors exhibiting < or >  = 18 methylated CpG sites (the cut-point for stratification represents the median of the distribution among MSP-positive tumors) was also highly divergent (*p* = 0.002; Fig. 3b); the survival curve of MSP-positive tumors lied in between that of tumors with < 18 (median OS: 17.1 months; 54 patients) or >  = 18 (median OS: 26.2 months; 59 patients) methylated CpG sites. Baseline patients’ characteristics did not significantly differ between the subgroups of MSP-positive tumors with < or >  = 18 methylated CpG sites. Stratification of MSP-negative tumors was not possible analogously since the overall median number of methylation CpG sites was too low in this group. All clusters (as displayed in Fig. 1c) were involved to a various degree in either of the prognostic subgroups; none of the clusters per se was associated with a favourable outcome (data not shown).

We plotted martingale residuals (Y-axis) versus the accumulated number of methylated CpG sites (X-axis; see Additional file 1: Fig. S1) to analyze whether the effect of the number of methylated CpG sites on the hazard ratio is linear and found the local linear regression (locally weighted scatterplot smoothing, LOWESS) curve to be linear in decrease pointing towards a linear effect. All of the analyzed CpG sites were significantly correlated with outcome; we did not find CpG sites or CpG subgroups with superior predictive impact within the investigated window of this series. Plotting martingale residuals versus the accumulated number of methylated CpG sites also revealed the same linear effect when respective CpG sites were classified as methylated in (1) case of a ratio of cytosine/thymine peak > 0.10 (orange, green and yellow labeling in Fig. 1b) or (2) case of a ratio of cytosine/thymine peak > 1.00 (green labeling in Fig. 1b) as displayed in Additional file 3: Fig. S3; however, clearest effects were seen when respective CpG sites were classified as methylated in case of a ratio of cytosine/thymine peak > 0.50 (green and yellow labeling in Fig. 1b).

Calculating the local linear regression (LOWESS) curves also revealed a linear effect of the number of methylated CpG sites on the hazard ratio for survival in both the subgroup of patients undergoing biopsy (n = 115) and that undergoing resection (n = 100) (see Additional file 2: Fig. S2); adjusted OS for those MSP-positive tumors exhibiting < or >  = 18 methylated CpG sites was divergent (resection: p = 0.02; biopsy: 0.04) and flanked the survival curve of MSP-positive tumors in both subgroups (see Additional file 4: Fig. S4).

**Fig. 4:**
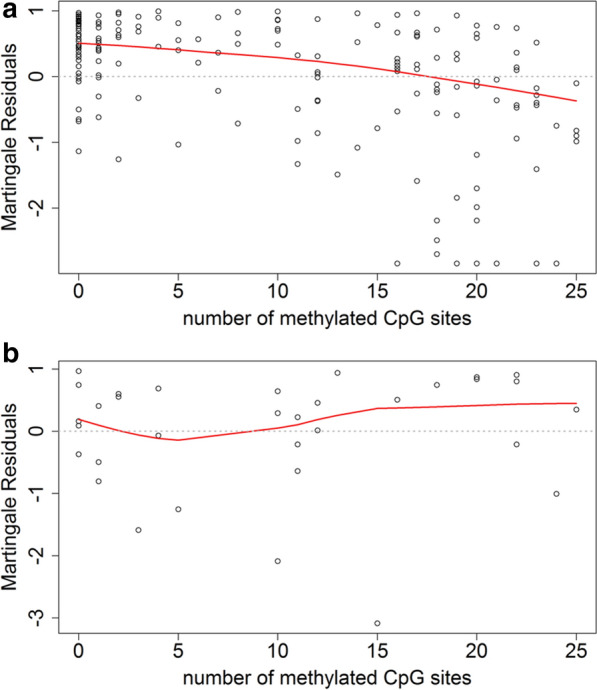
Effect of the number of ‘methylated’ CpG sites on the hazard of mortality: the patients’ ‘methylated’ CpG sites were plotted against the patients’ martingale residuals of the model without the variable “methylated CpG sites”. The red line shows the corresponding LOWESS (locally weighted scatterplot smoothing) curve and gives a hint for the true effect of the number of ‘methylated’ CpG sites on the hazard of mortality for glioblastoma patients with survival of at least three months and (**a**) at least one (n = 170) or (**b**) no (n = 33) adjuvant TMZ cycle after XRT/TMZ

When analyzing the subgroup of patients with OS > 3 months that received at least one adjuvant TMZ cycle after RT/TMZ (n = 170) in a landmark analysis, we found similar results as compared to the overall cohort: plotting martingale residuals versus the accumulated number of methylated CpG sites resulted in a LOWESS that was considered approximately linear in decrease (i.e. linear effect; see Fig. 4); survival for those MSP-positive tumors exhibiting < or >  = 18 methylated CpG sites was also highly divergent (*p* < 0.01) and flanked the survival curve of MSP-positive tumors (data not shown). For those patients with OS > 3 months that did not receive any adjuvant TMZ after RT/TMZ due to precedent progressive disease (n = 33), no correlation of the accumulated number of methylated CpG sites with survival could be seen.

### Post-recurrence survival

Median KPS at timepoint of progression was 70. For treatment of progression, 42 patients were primarily re-exposed to TMZ. 10 patients primarily underwent re-radiation, 18 patients re-OTR, 4 patients re-OTR combined with following re-radiation, 18 patients received stereotactic interstitial brachytherapy, 11 patients 5-aminolevulinic acid photodynamic therapy, and 5 patients bevacizumab; 42 of these 66 patients were re-exposed to TMZ afterwards, while 2 patients received bevacizumab and 22 patients best supportive care afterwards. 107 patients received best supportive care solely at the timepoint of progression.

Median overall PRS was 5.5 months, while median PRS for MSP-positive (MSP-negative) tumors was 6.6 (4.8) months (*p* = 0.002). When plotting martingale residuals versus the accumulated number of methylated CpG sites, we found the LOWESS curve to be linear only in the 88 patients with re-exposure to TMZ for post-progression treatment; in the remaining patients, we could not detect a similar linear effect of the number of methylated CpG sites on the hazard ratio for PRS (see Additional file 4: Fig. S4).

## Discussion

Clinical *MGMT* promoter status testing often relies on a very limited number of CpG sites within the *MGMT* promoter region and is reported categorially either as ‘methylated’ or ‘unmethylated’ [2, 19]. Such approaches, however, may miss the potential role of the patient’s individual CpG methylation pattern, quantitative differences in methylation, and cases of “grey zone methylation” as recently proposed [13, 24]. Based on a large uniform cohort of 215 glioblastoma patients, we applied MSP and Sseq testing in parallel and evaluated the results independently. Since Sseq enables analysis of the individual methylation status of 25 single CpG sites (including those 9 CpG sites covered by MSP) located in the DMR-2 island and downstream [2], we herein outline the role of the individual CpG site methylation profile on outcome among such patients (Additional file 5: Table S1; Additional file 6: Table S2).

We found that the cumulative number of methylated CpG sites within the *MGMT* promoter region is correlated with outcome. In dependence of the number of methylated CpG sites, adjusted OS and PFS were predicted to increase in a stepwise fashion. These findings might explain survival advantages among so called ‘unmethylated’ but also ‘methylated’ glioblastomas after TMZ (as determined per MSP). When looking on the martingale residuals of the model, a linear positive association between the number of methylated CpG sites and increased survival (OS or PFS) was demonstrated in the overall cohort. Subgroup analyses revealed that this linear association was present whenever glioblastoma patients were exposed to at least one adjuvant TMZ cycle in both primary situations after initial combined chemoradiotherapy (with regard to OS and PFS) as well as recurrent situations (with regard to PRS)—regardless of the initial surgical procedure for histological assessment (OTR vs. biopsy). No comparable association was seen in those patients without adjuvant resp. recurrent TMZ exposition.

To provide an easy to use tool for clinical practice, we additionally present a two-scaled survival model for MSP-positive tumors in the overall cohort using the median number of methylated CpG sites as cutoff value (< vs. >  = 18 methylated CpG sites) which allowed for predictive substratification of patients that are commonly summarized to be ‘methylated’ in terms of standard MSP-testing. Substratification might be particularly relevant when it comes to patients’ guidance by estimating prognosis or treatment decisions beyond standard treatment regimens. For instance, upfront TMZ compared to resection plus conventional chemoradiotherapy among the elderly subpopulation might be a more promising approach for MSP-positive tumors exhibiting a larger number of methylated CpGs. Whether this holds true in prospective cohorts remains to be shown, and no definitive treatment recommendations can be made based upon our retrospective study. However, such a hypothesis will need to be considered in future studies.

Recent studies have focused on substratification of glioblastoma patients by determining appropriate cutoffs and safety margins to distinguish “grey zone” methylation from truly “unmethylated” and truly “methylated” glioblastomas using quantitative MSP and/or pyrosequencing assay analyses. It was demonstrated that the extent of methylation impacts prognosis and enables a three scaled predictive model including a small tumor subgroup exhibiting “grey zone” methylation with a slightly significantly better prognosis than truly “unmethylated” glioblastomas [13, 24]. However, such studies might be of limited explanatory power since individual CpG methylation patterns were not addressed and stratificational differences in the “truly/highly methylated” group were not analyzed, whereas we did find such differences. Of note, in quantitative MSP and pyrosequencing assay analyses, the level of methylation measured is directly influenced by the tumor cell content of the tissue as only an average value is provided, whereas counting the number of CpG sites with a methylation level above a suitable threshold value (e.g. C/T-peak ratio ≥ 0.50) for a larger number of CpG sites, as described here, provides a quantitative readout which is robust to the admixture of some non-tumor cells. Particularly in the era of precision medicine, an increasing body of data supports the demand for more refined techniques for MGMT promoter methylation testing, allowing for a more precise substratification than a two- or three-scaled predictive model, and the Sseq approach, as described in this study, might be a viable option.

Of note, it remains to be shown whether our findings on the correlation between outcome and number of methylated CpG sites will hold true when tumors are graded according to potential future classification systems. In the present study, glioblastoma WHO grade IV was defined by histopathological findings based on the WHO 2016 classification. However, future studies may need to rest their definition of WHO grade on molecular findings. According to the most recent cIMPACT-NOW update, diffuse astrocytomas without IDH mutation formerly assigned to WHO grade II or III might be denoted as WHO grade IV in the presence of specific genetic alternations [18].

In this study, hierarchical clustering identified three distinct clusters of CpG sites with significantly different methylation rates suggesting a site-dependent methylation propagation. Moreover, the correlative matrix of hierarchical clustering uncovered a neighborhood-dependent methylation propagation, i.e. the methylation status of a given CpG site usually matched that of their neighbored CpG sites to some degree. Accordingly, the methylation status of the CpG sites was shown to be inter-correlated within the three distinct clusters and to a lesser degree also throughout different clusters. These correlative distributions, however, overlapped and did not allow accurate extrapolation of methylation levels from one subgroup to another one; therefore analysis of the individual MGMT promoter methylation status cannot be restricted to a single cluster. CpG site- and CpG neighborhood-dependent methylation propagation might have contributed to the observed methylation heterogeneity among MSP-positive tumors. Recently published Bayesian inference modelling of DNA methylation propagation at the MGMT promoter supports the concept of CpG site- and CpG neighborhood-dependent heterogeneous methylation propagation [4].

We did not find CpG hot-spots or subgroups with superior predictive impact within the investigated window of this series. All 25 CpG sites analyzed by Sseq were significantly correlated with outcome. Our findings strongly point to an inter-correlated predictive network of methylated CpG-sites varying among tumors. Studies speculating on the superior predictive impact of a single CpG site on the basis of more or less transcriptional silencing may have not controlled for the effects of inter-correlations in the CpG network of the promoter and other factors influencing MGMT silencing such as histone demethylation or transcript elongation by alternative polyadenylation and miRNA targeting [1, 9, 19, 25]. Future studies in prospective cohorts are warranted to determine whether there might be an association of individual CpG sites and outcome, however, our study seems not be in support of such a hypothesis.

## Conclusions

Collectively, extent of MGMT promoter methylation as determined by number of methylated CpG sites appears to correlate with outcome in a linear fashion. Sseq seems to be feasible in daily clinical practice to extensively analyse a large number of CpG sites within the DMR-2 island. Of note, this method provides a fast turnaround time of only 2–3 days, the amount of tissue/DNA required is small [compared e.g. to next-generation-sequencing (NGS)], standardized analysis provides high inter-rater reliability, and the technological infrastructure required is available at low costs worldwide. An up-front analysis of the individual GpC site methylation status might therefore help to improve the prognostic and predictive stratification of glioblastoma patients, which can be used for more precise prognostic and therapeutic concepts than conventional testing.

## Supplementary Information

**Additional file 1: Supplementary Fig. S1.** Effect of the number of ‘methylated’ CpG sites on the hazard of mortality: For 215 glioblastoma patients, the patients' ‘methylated’ CpG sites were plotted against the patients' martingale residuals of the model without the variable "methylated CpG sites". The red line shows the corresponding LOWESS (locally weighted scatterplot smoothing) curve and gives a hint that the true effect of the number of ‘methylated’ CpG sites on the hazard of mortality seems to be linear.

**Additional file 2: Supplementary Fig. S2.** Effect of the number of ‘methylated’ CpG sites on the hazard of mortality: the patients' ‘methylated’ CpG sites were plotted against the patients' martingale residuals of the model without the variable "methylated CpG sites". The red line shows the corresponding LOWESS (locally weighted scatterplot) curve and gives a hint for the true effect of the number of ‘methylated’ CpG sites on the hazard of mortality for the subgroups of glioblastoma patients undergoing **A**) biopsy (n = 115) or **B**) OTR (n = 100).

**Additional file 3: Supplementary Fig. S3.** Effect of the number of ‘methylated’ CpG sites on the hazard of mortality in 215 glioblastoma patients: the patients' ‘methylated’ CpG sites were plotted against the patients' martingale residuals of the model without the variable "methylated CpG sites". The red line shows the corresponding LOWESS (locally weighted scatterplot smoothing) curve and gives a hint for the true effect of the number of ‘methylated’ CpG sites on the hazard of mortality in case of respective CpG sites were classified as ‘methylated’ by a ratio of cytosine/thymine peak **A**) >0.10, **B**) >0.50 and **C**) >1.00.

**Additional file 4: Supplementary Fig. S4.** Effect of the number of ‘methylated’ CpG sites on the hazard of post-recurrence mortality: the patients' ‘methylated’ CpG sites were plotted against the patients' martingale residuals of the model without the variable "methylated CpG sites". The red line shows the corresponding LOWESS (locally weighted scatterplot) curve and gives a hint for the true effect of the number of ‘methylated’ CpG sites on the hazard of mortality for glioblastoma patients with post-recurrence treatment **A**) with (n = 88) and **B**) without (n = 120) TMZ re-exposition.

**Additional file 5: Supplemetary Table S1.** Survival data in different subgroups.

**Additional file 6: Supplemetary Table S2.** Study population.

## Data Availability

The datasets used and/or analysed during the current study available from the corresponding author on reasonable request.

## Abbreviations

18F-FET PET: *O*-(2-[18F]fluoroethyl)-1-tyrosine positron emission tomography
bp: Base pair
CI: Confidence interval
CpG: Cytosine-Guanine dinucleotide
CRAN: Comprehensive R Archive Network
C/T: Cytosine/thymine
DMR-2: Differentially methylated region-2
DNA: Deoxyribonucleic acid
GTR: Gross-total tumor resection
IDH1/2: Isocitrate dehydrogenase 1/2
IRB: Institutional Review Board
KPS: Karnofsky performance status
LOWESS: Locally weighted scatterplot smoothing
MGMT: *O*-6-Methylguanine-DNA methyltransferase
MRI: Magnetic resonance imaging
MSP: Methylation-specific polymerase-chain-reaction
NGS: Next-generation-sequencing
PFS: Progression-free survival
PRS: Post-recurrence survival
RANO: Response assessment in neuro-oncology
RT: Radiotherapy
OS: Overall survival
OTR: Open tumor resection
STR: Subtotal tumor resection
Sseq: Sanger sequencing
TMZ: Temozolomide
WHO: World Health Organization
