# Supplementary figures and images for: The number of methylated CpG sites within the MGMT promoter region linearly correlates with outcome in glioblastoma receiving alkylating agents
Source: Acta Neuropathol Commun. 2021 Mar 4;9:35. doi: 10.1186/s40478-021-01134-5 (PMC7934240; doi:10.1186/s40478-021-01134-5)

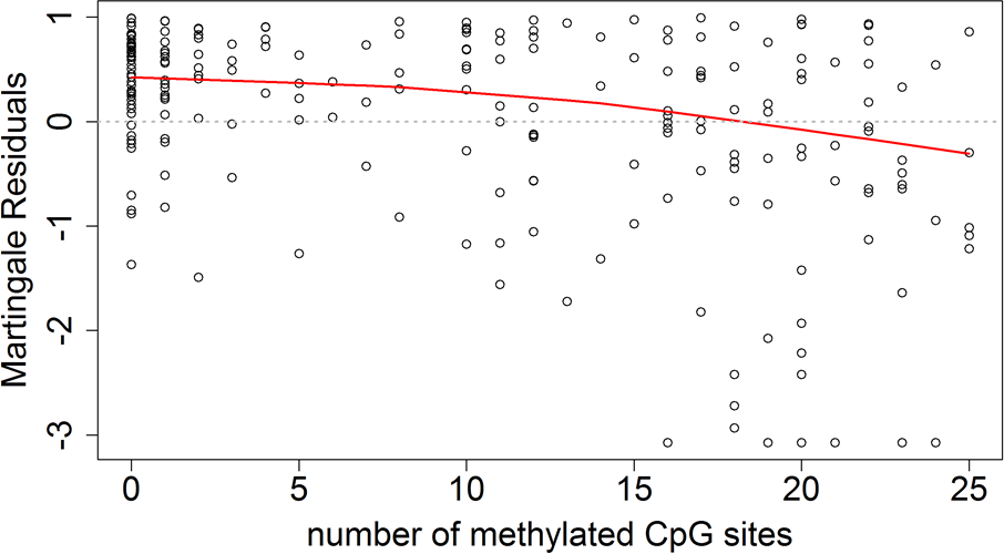

Supplement: Supplementary file 1 — Additional file 1: Supplementary Fig. S1. Effect of the number of ‘methylated’ CpG sites on the hazard of mortality: For 215 glioblastoma patients, the patients' ‘methylated’ CpG sites were plotted against the patients' martingale residuals of the model without the variable "methylated CpG sites". The red line shows the corresponding LOWESS (locally weighted scatterplot smoothing) curve and gives a hint that the true effect of the number of ‘methylated’ CpG sites on the hazard of mortality seems to be linear. [file 40478_2021_1134_MOESM1_ESM.tif]

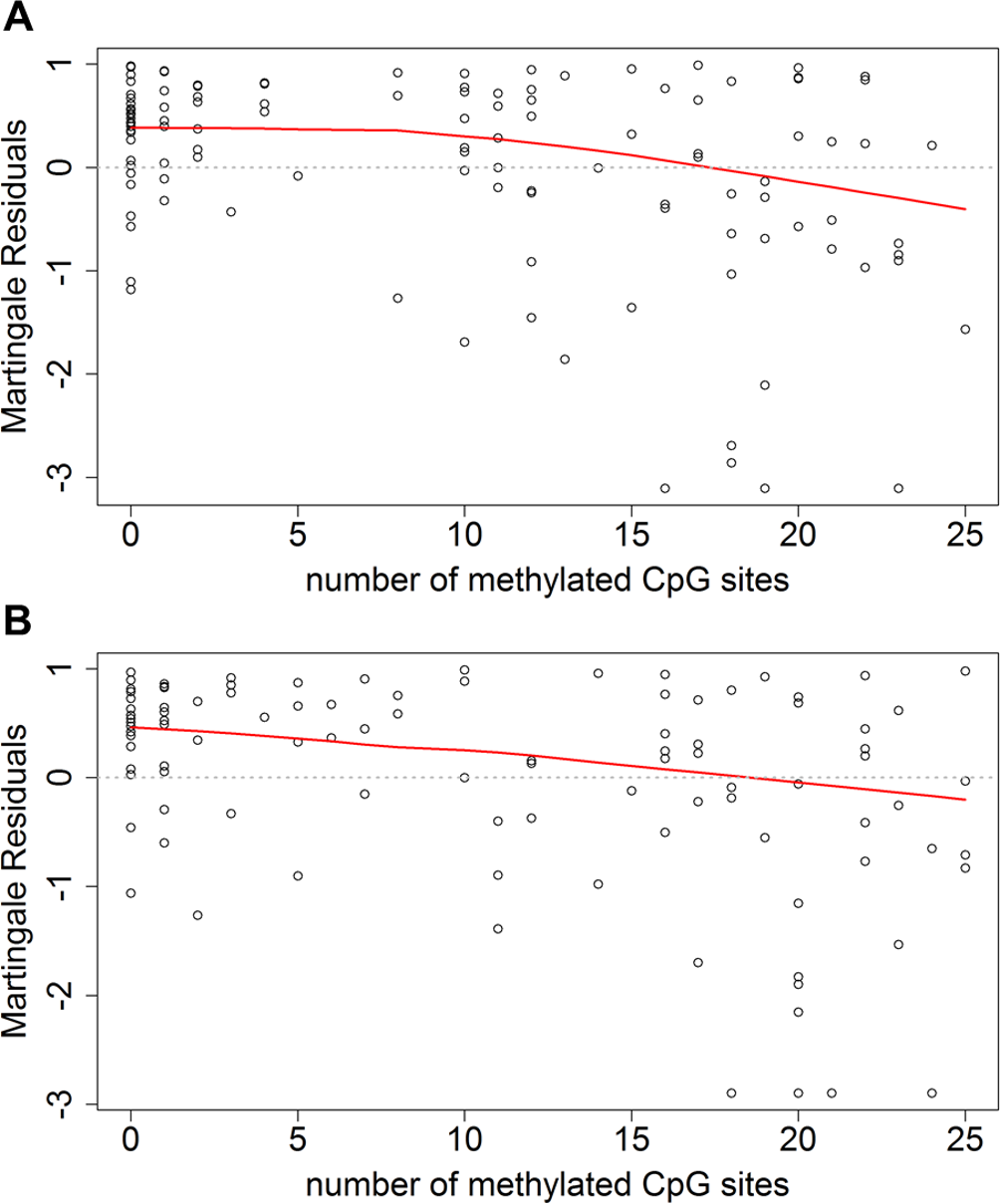

Supplement: Supplementary file 2 — Additional file 2: Supplementary Fig. S2. Effect of the number of ‘methylated’ CpG sites on the hazard of mortality: the patients' ‘methylated’ CpG sites were plotted against the patients' martingale residuals of the model without the variable "methylated CpG sites". The red line shows the corresponding LOWESS (locally weighted scatterplot) curve and gives a hint for the true effect of the number of ‘methylated’ CpG sites on the hazard of mortality for the subgroups of glioblastoma patients undergoing A) biopsy (n = 115) or B) OTR (n = 100). [file 40478_2021_1134_MOESM2_ESM.tif]

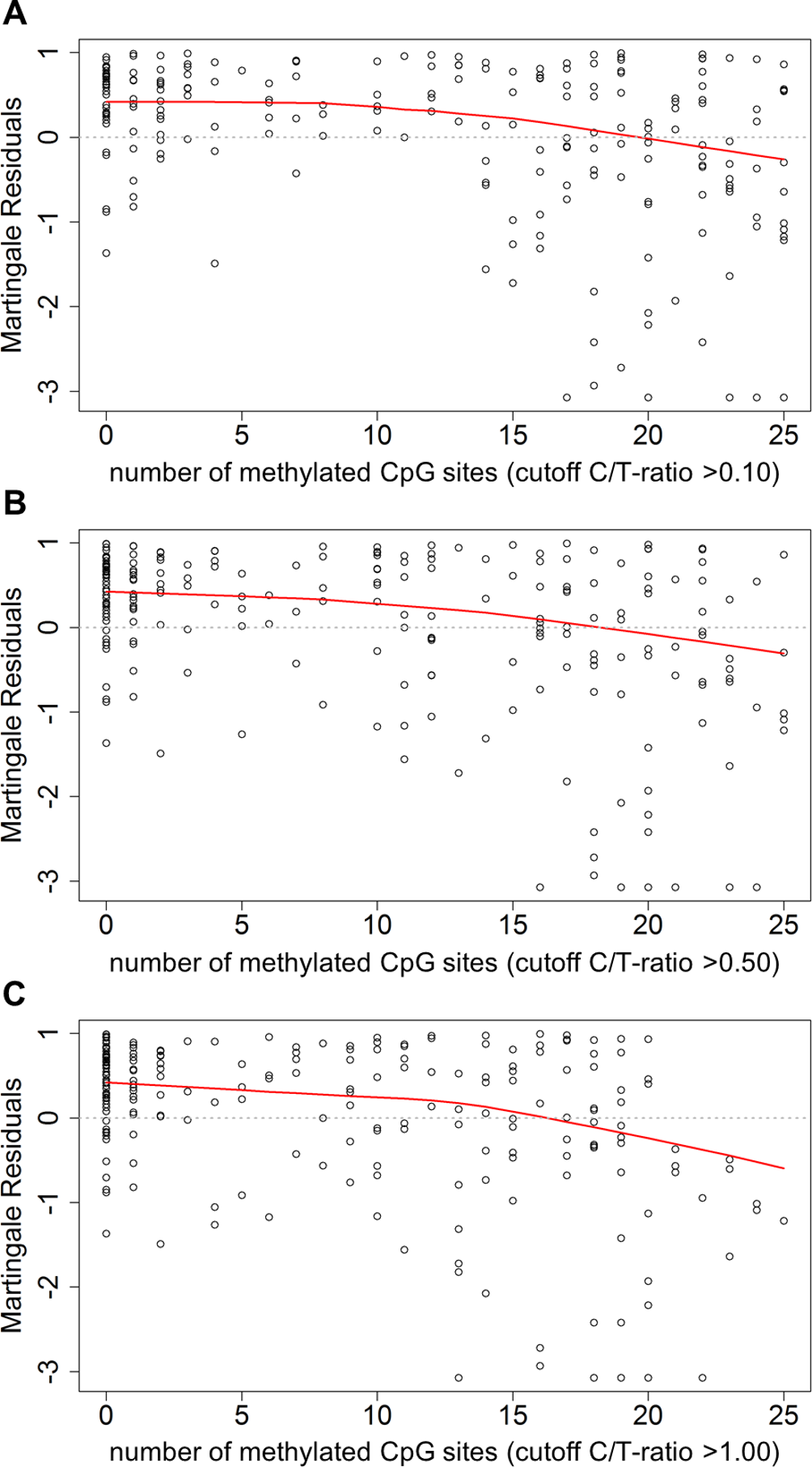

Supplement: Supplementary file 3 — Additional file 3: Supplementary Fig. S3. Effect of the number of ‘methylated’ CpG sites on the hazard of mortality in 215 glioblastoma patients: the patients' ‘methylated’ CpG sites were plotted against the patients' martingale residuals of the model without the variable "methylated CpG sites". The red line shows the corresponding LOWESS (locally weighted scatterplot smoothing) curve and gives a hint for the true effect of the number of ‘methylated’ CpG sites on the hazard of mortality in case of respective CpG sites were classified as ‘methylated’ by a ratio of cytosine/thymine peak A) >0.10, B) >0.50 and C) >1.00. [file 40478_2021_1134_MOESM3_ESM.tif]

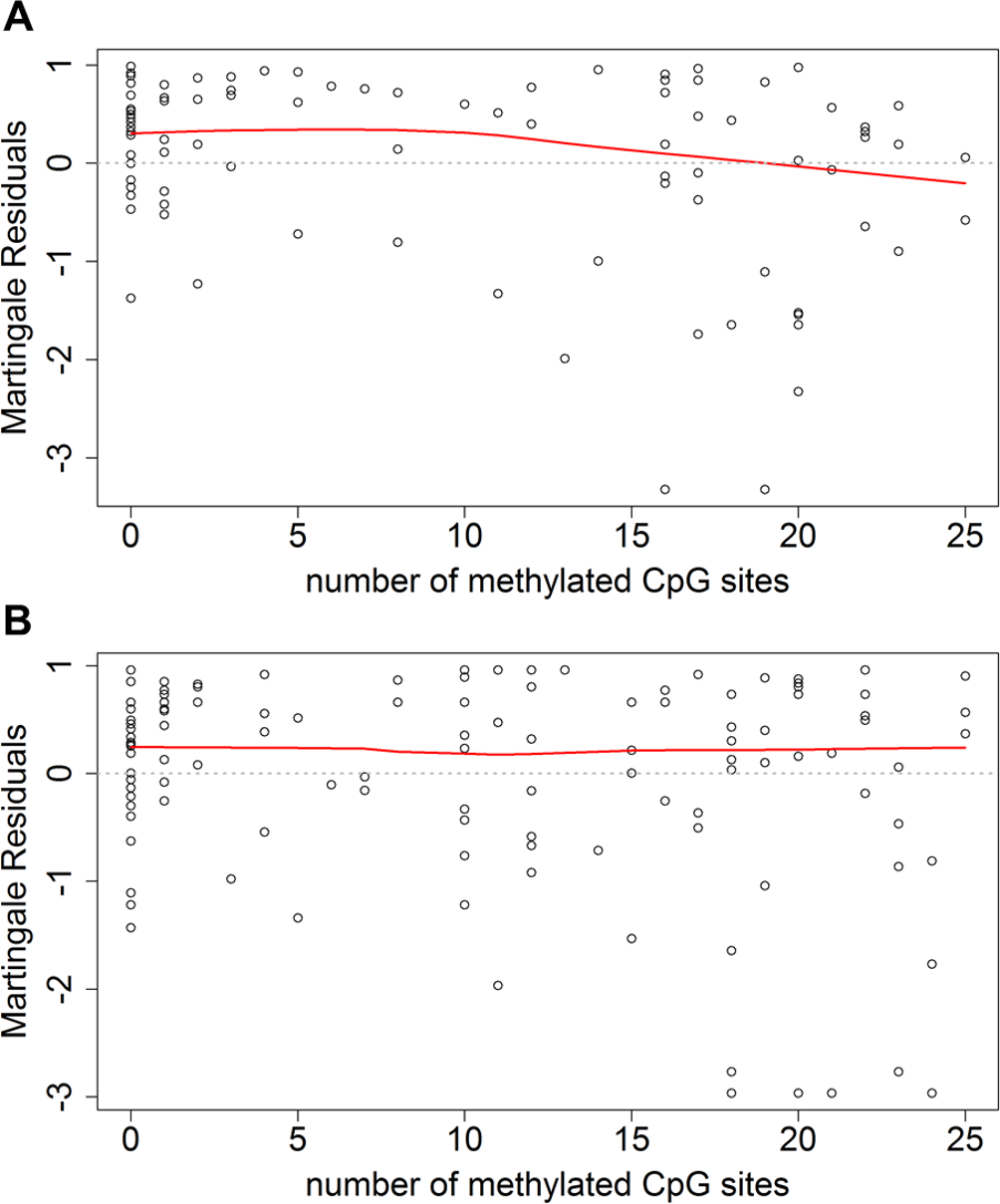

Supplement: Supplementary file 4 — Additional file 4: Supplementary Fig. S4. Effect of the number of ‘methylated’ CpG sites on the hazard of post-recurrence mortality: the patients' ‘methylated’ CpG sites were plotted against the patients' martingale residuals of the model without the variable "methylated CpG sites". The red line shows the corresponding LOWESS (locally weighted scatterplot) curve and gives a hint for the true effect of the number of ‘methylated’ CpG sites on the hazard of mortality for glioblastoma patients with post-recurrence treatment A) with (n = 88) and B) without (n = 120) TMZ re-exposition. [file 40478_2021_1134_MOESM4_ESM.tif]
