# Supplementary material for: The number of methylated CpG sites within the MGMT promoter region linearly correlates with outcome in glioblastoma receiving alkylating agents
Source: Acta Neuropathol Commun. 2021 Mar 4;9:35. doi: 10.1186/s40478-021-01134-5 (PMC7934240; doi:10.1186/s40478-021-01134-5)
Supplement: Supplementary file 5 — Additional file 5: Supplemetary Table S1. Survival data in different subgroups. [file 40478_2021_1134_MOESM5_ESM.docx]

| **SUPPLEMENTARY TABLE 1: Survival data in different subgroups** | | | | |
| --- | --- | --- | --- | --- |
|  | **OTR**  (n = 100) | **Biopsy**  (n = 115) | **Lobar tumor**  (n = 139) | **Non-lobar tumor**  (n = 76) |
| **Progression-free survival** |  |  |  |  |
| **MSP negative**  n (%)  median (months) | 48 (48.0)  7.2 | 54 (47.0)  6.3 | 64 (46.0)  6.8 | 38 (50.0)  6.8 |
| **MSP positive**  n (%)  median (months) | 52 (52.0)  14.8 | 61 (53.0)  8.9 | 75 (54.0)  11.4 | 38 (50.0)  9.2 |
| **MSP positive** <18 meth. CpG-sites  no.  median (months) | 22 (22.0)  14.8 | 29 (25.2)  6.4 | 35 (25.2)  6.5 | 19 (25.0)  8.9 |
| **MSP positive** ≥18 meth. CpG-sites  n (%)  median (months) | 30 (30.0)  19.7 | 32 (27.8)  14.5 | 40 (28.8)  19.7 | 19 (25.0)  11.8 |
| **p-value**  MSP pos. vs. neg. | <0.01 | <0.01 | <0.01 | <0.01 |
| **p-value**  MSP pos. <18 vs. ≥18 meth. CpG-sites | 0.03 | <0.01 | <0.01 | 0.3 |
| **Overall survival** |  |  |  |  |
| **MSP negative**  n (%)  (months) | 48 (48.0)  15.4 | 54 (47.0)  9.0 | 64 (46.0)  12.8 | 38 (50.0)  11.2 |
| **MSP positive**  n (%)  median (months) | 52 (52.0)  26.6 | 61 (53.0)  14.3 | 75 (54.0)  21.7 | 38 (50.0)  18.5 |
| **MSP positive** <18 meth. CpG-sites  n (%)  median (months) | 22 (22.0)  21.7 | 29 (25.2)  11.7 | 35 (25.2)  14.6 | 19 (25.0)  19.2 |
| **MSP positive** ≥18 meth. CpG-sites  n (%)  median (months) | 30 (30.0)  28.5 | 32 (27.8)  23.7 | 40 (28.8)  28.6 | 19 (25.0)  16.7 |
| **p-value**  MSP pos. vs. neg. | <0.01 | <0.01 | <0.01 | <0.01 |
| **p-value**  MSP pos. <18 vs. ≥18 meth. CpG-sites | 0.02 | 0.04 | <0.01 | 0.8 |
|  | | | | |
