# Supplementary material for: The number of methylated CpG sites within the MGMT promoter region linearly correlates with outcome in glioblastoma receiving alkylating agents
Source: Acta Neuropathol Commun. 2021 Mar 4;9:35. doi: 10.1186/s40478-021-01134-5 (PMC7934240; doi:10.1186/s40478-021-01134-5)
Supplement: Supplementary file 6 — Additional file 6: Supplemetary Table S2. Study population. [file 40478_2021_1134_MOESM6_ESM.docx]

| **SUPPLEMENTARY TABLE 2: Study population** | |
| --- | --- |
| **Gender**  male/female, (n/n, %/%) | 142 (66.0) / 73 (34.0) |
| **Age (years)**  median (range) | 60 (17 - 86) |
| **KPS**  median (range) | 80 (40 - 100) |
| **Tumor side**  left/right (n/n, %/%) | 117 (54.4) / 98 (46.6) |
| **Tumor location**  lobar/deep^a^/multifocal (n/n/n, %/%/%) | 139 (64.7) / 20 (9.3) / 56 (26.0) |
| **MSP status**  positive/negative (n/n, %/%) | 113 (52.6) / 102 (47.4) |
| **Sanger sequence analysis**  number of methylated CpG sites (n, %)  0  1  2  3  4  5  6  7  8  9  10  11  12  13  14  15  16  17  18  19  20  21  22  23  24  25 | 43 (20.0)  20 (9.3)  11 (5.1)  5 (2.3)  5 (2.3)  5 (2.3)  2 (0.9)  3 (1.4)  6 (2.8)  0 (0.0)  11 (5.1)  9 (4.2)  14 (6.5)  2 (0.9)  3 (1.4)  4 (1.9)  10 (4.7)  9 (4.2)  10 (4.7)  7 (3.2)  13 (6.0)  4 (1.9)  10 (4.7)  7 (3.2)  3 (1.4)  5 (1.4) |
| **Surgical treatment**  GTR (n, %)  STR (n, %)  Biopsy (n, %) | 63 (29.3%)  37 (17.2%)  115 (53.5%) |
| **Treatment after surgery**  XRT/TMZ (n, %) | 215 (100.0) |
| **Adjuvant treatment**  TMZ (n, %)  no TMZ / only best supportive care | 120 (57.7)  88 (42.3) |
| **Post-recurrence treatment**  1^st^-line TMZ re-exposition (n, %)  re-radiation (n, %)  re-OTR (n, %)  re-OTR and re-radiation (n, %)  5-ALA photodynamic therapy (n, %)  1^st^-line bevacizumab (n, %)  2^nd^-line TMZ re-exposition (n, %)  2^nd^-line bevacizumab (n, %)  2^nd^-line best supportive care (n, %)  1^st^-line best supportive care | 42 (19.5)  10 (4.7)  18 (8.4)  4 (1.9)  11 (5.1)  5 (2.3)  44 (20.5)  2 (0.9)  22 (10.2)  107 (49.8) |
| **Median progression-free survival**  **(months)**  overall  OTR  biopsy  MSP negative  MSP positive  with ≥18 methylated CpG sites (Sseq)  with <18 methylated CpG sites (Sseq) | 7.9  9.8  6.9  6.8  10.6  15.6  6.7 |
| **Post-recurrence survival**  **(months)**  overall  OTR  biopsy  MSP negative  MSP positive  with ≥18 methylated CpG sites (Sseq)  with <18 methylated CpG sites (Sseq) | 5.5  7.8  3.1  4.8  6.6  7.5  5.8 |
| **Median overall survival**  **(months)**  overall  OTR  biopsy  MSP negative  MSP positive  with ≥18 methylated CpG sites (Sseq)  with <18 methylated CpG sites (Sseq) | 14.9  20.2  10.4  12.1  21.4  26.2  17.1 |
| ^a^ deep seated = not lobarly located (e.g. thalamus or basal ganglia) | |
